# Towards precision medicine for sepsis patients

**DOI:** 10.1186/s13054-016-1583-z

**Published:** 2017-01-12

**Authors:** Peter Pickkers, Matthijs Kox

**Affiliations:** 1 Department of Intensive Care Medicine, Radboud Institute for Molecular Life Sciences, Radboud University Medical Center, Internal mail 710, P.O. Box 9101, Nijmegen, 6500 HB The Netherlands; 2 Radboud Center for Infectious Diseases (RCI), Nijmegen, The Netherlands

**Keywords:** Sepsis, Hyperinflammation, Immune suppression, Cytokines, Precision medicine, Personalized, Markers, HLA-DR, PD-1, PD-L1

Over the last decade it has become clear that the immunological response and clinical course in sepsis patients is too complex to simply regard it as ‘hyperinflammation-induced organ failure’. In contrast to the previous belief that patients mainly suffer from an exaggerated pro-inflammatory response, it has now become evident that a pro- and anti-inflammatory response are mounted simultaneously [1, 2]. Whether pro-inflammation or anti-inflammation is the overruling immune response may differ between patients and evolve over time in an individual patient [2], and this may explain why all previous clinical studies in sepsis patients using interventions to attenuate the immune response are negative [3]. Greater appreciation for the role of the *dysregulated* immune response is represented in the new definition of sepsis [4], defining sepsis as a life-threatening organ dysfunction caused by a dysregulated host response to infection.

## One size does not fit all

Dozens of trials have convincingly demonstrated that inhibition of the immune response exerts no overall beneficial effects in the heterogeneous group of sepsis patients. Nevertheless, it is conceivable that a subgroup of patients may have benefited from inhibition of the immune response, but that in another subgroup of patients where immune suppression was the overriding immune dysregulation, no beneficial (or even detrimental) effects were evoked. Indeed, the interleukin (IL)-1 receptor blockade phase 3 trial published in 1997 [5] showed no effect of immune suppression with anakinra on mortality in patients suffering from severe sepsis. Based on new insights, a post-hoc analysis of this study performed 19 years later [6] identified that 5.6% of the study population presented with features of macrophage activation syndrome (sepsis with concurrent hepatobiliary dysfunction/disseminated intravascular coagulation). In this subgroup, mortality was 65% in the placebo-treated patients and 35% in the IL-1 receptor blockade patients (*p* = 0.0006) [6]. Naturally, this is a post-hoc analysis and no definitive conclusions can be drawn, but it does suggest that a specific subgroup of ‘hyperinflamed’ sepsis patients might benefit from inhibition of the immune response. The extent of this effect warrants further prospective study.

In contrast to this subgroup of patients that are hyperinflamed, observational data indicate that in a large proportion of sepsis patients immune suppression is the overriding immune dysregulation. For instance, sepsis patients are more likely develop infections with opportunistic bacteria or fungi [7] and up to 40% of patients show positive viral polymerase chain reactions (PCRs) indicating reactivation of latent viruses [8]. Furthermore, in a post-mortem study [9], profound suppression of immune cell function was found in tissue from patients that died of sepsis. Theoretically, pharmacological interventions aimed to stimulate recovery of this immune suppression might benefit this subgroup of patients.

Based on the above, it is clear that differentiation within sepsis patients is required to move this field forward, and immune phenotyping of sepsis patients may pave the way towards a more personalized approach, also called ‘precision medicine’.

## Determining the immune status of sepsis patients

Various ways to gauge the immune status of sepsis patients have been described [2], and many more will likely emerge in the following years as a consequence of progress in our insight of which pathways play a role in the development of immune suppression in sepsis patients. For example, recent evidence points towards an important role for defects in energy metabolism in the impaired function of immune cells of septic patients [10]. As such, it can be envisioned that profiling of cellular metabolism provides a snapshot of the current immunological state of a septic patient and may be used to guide treatment. Furthermore, the programmed death 1 (PD-1) pathway appears to be of particular relevance in sepsis-induced immune suppression, and expression of PD-L1 (the ligand for PD-1) on the cell surface of monocytes is associated with risk stratification and mortality in septic patients [11]. As ex vivo addition of antibodies against PD-1 or PD-L1 restored function of monocytes, neutrophils, T cells, and natural killer (NK) cells [12], the data from clinical studies in the field of oncology that show that treatment with anti-PD-1/PD-L1 antibodies is feasible and safe [13] is of interest to the sepsis field. When these compounds are tested in immune suppressed sepsis patients, patient group enrichment by determination of monocyte PD-L1 expression is clearly of utmost relevance. Several other compounds (including interferon (IFN)γ, granulocyte macrophage colony-stimulating factor (GM-CSF), and IL-7) that stimulate the immune system through various pathways have already shown promising results in preclinical studies, and some even in small patient studies. For a fair chance of clinical success, it is pivotal that we do not make the same mistake again by advocating the use of these compounds in all sepsis patients, but rather use them in an immune-suppressed subgroup.

Up to now, two assays to determine the immune status in sepsis patients have been studied most extensively: ex vivo lipopolysaccharide (LPS)-induced cytokine production by leukocytes, and monocytic human leukocyte antigen-D related (HLA-DR) expression. The LPS-stimulated whole blood functional assay provides insight in the actual cell function, while determination of monocyte HLA-DR expression is more specific and at the single cell level. Drewry et al. recently compared these two methods head-to-head [14]. In 83 patients, of which over 60% suffered from septic shock, more than a quarter of the patients developed a secondary infection and in total 30% died. Blood samples were collected at three time points (days 1–2, days 3–4, and days 6–8 after sepsis diagnosis) and results were related to the development of secondary infections and mortality. Their main finding is that monocytic HLA-DR expression is a more accurate predictor for secondary infections and mortality than ex vivo LPS-induced cytokine production, and in their paper they describe several explanations for their finding. The authors did not calculate specificity and sensitivity, as a specific threshold would have to be chosen to designate the test as being “positive” or “negative”. Other researchers [15] have used values <8000 antibodies/cell as the diagnostic threshold for immunosuppression to investigate the effect of immunostimulatory therapy. Also, although it is tempting to speculate that the change in HLA-DR expression results in an increased risk to develop secondary infections and is directly related to the impaired outcome in these patients, the authors wisely refrain from such an interpretation as it could also be an epiphenomenon: more severely ill patients might be more likely to develop a secondary infection and have a higher mortality independent of HLA-DR expression. Of interest, the change in HLA-DR expression that was most associated with the development of secondary infections occurred between days 1–2 to days 3–4 (median change in HLA-DR of −934 antibodies/cell; *p* = 0.054). As the median time to onset of secondary infection was 9.4 (interquartile range (IQR) 5.5 to 10.0) days, this indicates that the change in HLA-DR expression preceded the diagnosis of the secondary infection. It appears likely that HLA-DR expression may even be lower in the days immediately preceding the development of a secondary infection, but because of the limited number of patients evaluated in each group, the current study was underpowered to detect this. Ultimately, it may be more informative to assess multiple immunological markers concurrently or monitor changes in markers over time.

In conclusion, it is important to emphasize that the aim of the present study was not to investigate cause-effect relationships, as it remains unclear if a decrease in HLA-DR expression is a true predictor or merely an epiphenomenon. Nevertheless, it shows that HLA-DR expression is more accurate in predicting secondary infections and death in sepsis patients than LPS-induced cytokine production. This indicates that HLA-DR expression may be used to identify whether or not a sepsis patient is in the immunosuppressive phase of their disease. This is of utmost importance, as it has become clear that undifferentiated immunomodulatory therapy in sepsis patients is bound to fail. It appears plausible that some patients may benefit from inhibition of the immune response, while others may benefit from stimulation of the immune response. Immunostimulatory treatment should only be offered to those patients who suffer from a suppressed immune system and, for now, monocytic HLA-DR expression is the optimal marker to determine this.

## Abbreviations

GM-CSF: Granulocyte macrophage colony-stimulating factor
HLA-DR: Human leukocyte antigen-D related
IFN: Interferon
IL: Interleukin
IQR: Interquartile range
LPS: Lipopolysaccharide
NK: Natural killer
PCR: Polymerase chain reaction
PD-1: Programmed death 1
PD-L1: Programmed death-ligand 1
